# Energy Harvesting in a System with a Two-Stage Flexible Cantilever Beam

**DOI:** 10.3390/s22197399

**Published:** 2022-09-28

**Authors:** Jerzy Margielewicz, Damian Gąska, Grzegorz Litak, Piotr Wolszczak, Shengxi Zhou

**Affiliations:** 1 Faculty of Transport and Aviation Engineering, Silesian University of Technology, 40-019 Katowice, Poland; 2 Faculty of Mechanical Engineering, Lublin University of Technology, 20-618 Lublin, Poland; 3 School of Aeronautics, Northwestern Polytechnical University, Xi’an 710072, China

**Keywords:** bifurcations, lyapunov exponent, periodicity, energy efficiency, chaos

## Abstract

The subject of the research contained in this paper is a new design solution for an energy harvesting system resulting from the combination of a quasi-zero-stiffness energy harvester and a two-stage flexible cantilever beam. Numerical tests were divided into two main parts-analysis of the dynamics of the system due to periodic, quasiperiodic, and chaotic solutions and the efficiency of energy generation. The results of numerical simulations were limited to zero initial conditions as they are the natural position of the static equilibrium. The article compares the energy efficiency for the selected range of the dimensionless excitation frequency. For this purpose, three cases of piezoelectric mounting were analyzed-only on the first stage of the beam, on the second and both stages. The analysis has been carried out with the use of diagrams showing difference of the effective values of the voltage induced on the piezoelectric electrodes. The results indicate that for effective energy harvesting, it is advisable to attach piezoelectric energy transducers to each step of the beam despite possible asynchronous vibrations.

## 1. Introduction

The use of electricity in all spheres of human life is constantly increasing. At the same time, the challenges of today’s world related to ecology, certain limitations of renewable sources and depleted fossil fuels have resulted in the search for solutions allowing for more efficient use of available energy, in particular when we are dealing with phenomena that are even common in technology, such as energy lost due to friction and heat release, acoustic effects or vibration propagation and related unfavorable phenomena. All of them cause a loss of energy from the system which, if properly processed, could become useful to power small electronic device sensors [1,2,3]. We can use energy harvesters to process such energy [4]. They are not sources that generate large amounts of energy, therefore researchers constantly work on increasing their efficiency [5,6]. In laboratories and simulations, mostly harmonic [7], stochastic methods [8] or their combinations are used to test the mechanical energy harvesters [9]. In other applications, intermitted or time-limited excitations are available, other than simple ideal harmonic excitation or stochastic methods [10,11]. This is especially useful for real vibration sources of energy such as ocean waves, wind, passing trains and walking persons [12,13,14,15].

From a technical point of view, the design of the vast majority of energy harvesters for vibrating mechanical systems is based on a flexible cantilever beam resonator coupled to a piezoelectric transducer [1,7,16]. Unfortunately, the performance of such a system has a limited applicability to the resonance frequency of the beam. On the other hand, permanent magnets inducing nonlinear forces are used to modify the form potential of nonlinear harvester systems [17] to go beyond single-resonance-frequency working conditions. Namely, magnets modify the stiffness of the system and lead to a multi-well potential which leads to a wider effective frequency range and additional solutions present in the nonlinear system [18]. As all the solutions have their own frequencies for the maximum outputs (including new resonances) nonlinear vibration energy harvesters are robust against frequency variability of vibration sources [19,20]. It is a feature helpful for real applications using ambient vibration sources with uncertain parameters [21,22].

In addition to magnets, as elements modifying the potential barrier, there are also used, inter alia, elastic and damping elements, such as springs [22,23] or various beam structures, additional degrees of freedom, bumpers etc. [24,25]. Such structures have both advantages and disadvantages. They allow for a fairly simple modification of parameters, but from an engineering point of view, they can be a challenge due to the more complicated structure. The nonlinear characteristics significantly increase the efficiency of energy harvesting [7,8] through the distortion of the resonance region [26] and the occurrence of additional solutions. Namely, resonant and non-resonant solutions can appear in the strongly inclined resonance curve. Additionally, sub- and super-harmonic solutions can appear in a wide frequency range [27,28]. This is important to the design of self-powered sensors in the presence of a variable source. As elements transforming mechanical energy into electricity, various transducers are used, the most common of which are piezoelectric ones [29,30] due to their simple structure and high energy density [31,32]. The nonlinear monostable or bistable mechanical structures designed for effective vibration insulation and harvesting may include X-shaped springs [33], K-shaped springs [34] bio-inspired structures [35,36,37], and different combination of linkages and spring.

Nowadays, multi-stable vibration energy harvesters (MEHs) are of particular interest to researchers due to their wide application possibilities [38] and nonlinear properties, and thus better energy harvesting efficiency. For this reason, newer and more efficient designs are analyzed, which allow the conversion of mechanical vibrations into usable energy to power small sensors and electronic devices [39,40]. The aim is to eliminate batteries or extend their lives, which will allow the use of such sensors, for example in hard-to-reach places, and will supply these sensors. Basic MEHs are bistable with two stable positions and one unstable position. They have been widely tested, resulting in various proposed designs [7,8,26,27,41,42,43]. In addition, theoretical models and experimental investigations of a broadband piezoelectric-based vibration energy harvesters with tri-stable functions of potential barriers were proposed and developed [44,45,46,47]. To enhance the performance of the bistable and tri-stable energy harvesters, quad-stable and penta-stable energy harvesters were designed by adding more fixed magnets presented in [48,49] or with combined nonlinearity of cantilever-surface contact and magnetoelasticity [50].

It is worth noticing that the limit of increasing multistability naturally leads to decreasing the individual potential barriers. In that context, the quasi-zero stiffness case is a simplified realization of multiple equilibria coming from the flattened series of many potential wells [48,49] or case-characterized by the potential at the bifurcation point between monostable and bistable potentials [51] with respect to the control parameter. Such a solution was also proposed and proven to be useful for energy harvesting [52,53].

On the other hand, an additional spring component can work as displacement amplificator acting effectively for the bistable harvester, as recently described by Liu at al. [54]. In our case, the extra spring in the harvester system coincides with the first stage of the proposed composite beam. Consequently, the mechanical resonator system of the energy harvester is effectively approximated by a 2DOF system. In this way, the above-mentioned design solutions and examples of the use of 2DOF quasi-zero stiffness energy harvesters were the motivation to analyze a new solution, which is an extension of single DOF structures proposed and published by the same authors [52,53]. Note also that the subject of the model research contained in this paper is a new design of the energy generation system with a two-stage flexible cantilever beam and a quasi-zero stiffness system. In Section 2 we proposed a model and method of operation, and in Section 3 we compared its energy efficiency with the quasi-zero-stiffness energy harvester (QZEH), presenting the results in the form of effective voltage values.

## 2. Mathematical Model Formulation

**Figure 1:**
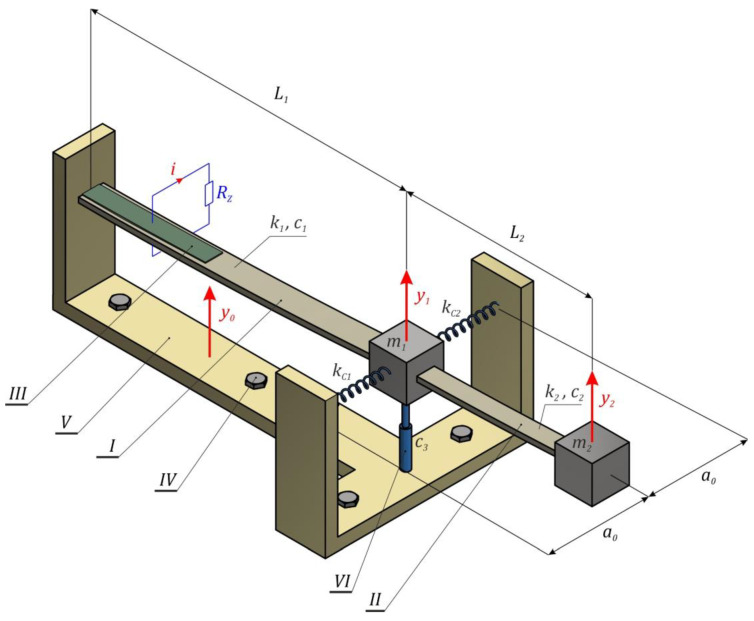
Schematic diagram of the energy harvester with a two-stage cantilever beam for proposed inertial energy harvesting.

The subject of the model tests presented in this paper is the energy harvesting system with a two-stage composite flexible cantilever beam (Figure 1). A significant feature of such a design solution is the possibility of designing them both on a micro and macro scale. The considered energy harvester consists of flexible beams *I* and *II*, the ends of which are loaded with inertial elements *m_i_*. The inertial element *m*_1_ is supported in the joint by a mini shock absorber *VI*. Piezoelectric elements *III* were glued to the flat surfaces of the first stage of the beam *I*, which under the influence of an external dynamic excitation undergoes elastic deformation, as a result of which an electric charge is induced on the piezoelectric electrodes. The elastic elements of the analyzed design solution of the system were mounted in a rigid, non-deformable *V* frame, which was screwed by means of *IV* bolts to the mechanically vibrating object.

Based on the formulated phenomenological model, its mathematical representation was derived. During its derivation, it was assumed that the mechanical properties of the mini shock absorber were represented by the linear damping characteristic. Moreover, when formulating the design assumptions, the same stiffness of the *k_Ci_* compensation springs was assumed. To estimate the elastic properties characterizing the individual degrees of a cantilever beam, it is possible to identify it based on the equation of the elastic curve. However, the dissipation properties are identified on the logarithmic decrement [55]. Additionally, we assume that the tested design of the energy harvesting system is influenced by mechanical vibrations described by the harmonic function: $y_{0}=Asin\left(\omega_{W}t\right)$. To derive the set of mechanical equations (see below Equation (1)) we balance the forces caused by linear springs of stiffness *k*_1_ and *k*_2_, linear dampers of camping coefficients *c*_1_, *c*_2,_ and *c*_3_ and use the inertial masses *m*_1_ and *m*_2_ in the inertial system (Figure 1). Additionally, suspension (compensation) springs with the effective stiffness *k_z_* = 2*k*_ci_ were included. Note that the spring will generate nonlinear force with respect to the displacement *y*_1_ as the spring deformation is following the diagonal suspended on the rectangular of *a*_0_ and *y*_1_ sides. The transversal orientation of these springs with respect to the displacement direction of *y*_1_ produces the geometrical nonlinearity during motion. 

Bearing in mind the adopted model and simplifying assumptions, the differential equations of motion were written, in which the relative coordinates were taken into account: $q_{1}=y_{1}-y_{0}$ and $q_{2}=y_{2}-y_{1}$:

(1) $$\left\{\begin{array}{c} m_{1}\frac{d^{2}q_{1}}{dt^{2}}+c_{1}\frac{dq_{1}}{dt}+c_{3}\frac{dq_{1}}{dt}-c_{2}\frac{dq_{2}}{dt}+k_{1}q_{1}+k_{2}q_{2}+k_{Z}\left(1-\frac{a_{0}}{\sqrt{a_{0}^{2}+q_{1}^{2}}}\right)q_{1}+\\ +k_{P}u=-m_{1}\frac{d^{2}y_{0}}{dt^{2}},\\ m_{2}\frac{d^{2}q_{2}}{dt^{2}}+c_{2}\frac{dq_{2}}{dt}+k_{2}q_{2}=-m_{2}\frac{d^{2}y_{0}}{dt^{2}}-m_{2}\frac{d^{2}q_{1}}{dt^{2}},\\ C_{P}\frac{du}{dt}+\frac{1}{R_{Z}}u-k_{P}\frac{dq_{1}}{dt}=0. \end{array}\right.$$

The electrical subsystem coefficients appearing in the mathematical model (1) represent, respectively: constant *k_P_*, piezoelectric capacity *C_P_*. The parameter *R_Z_*, on the other hand, represents the equivalent resistance of the load and the electric circuit. In order to efficiently carry out computer simulations, a dimensionless mathematical model was derived. Such a representation is the formal basis for carrying out quantitative and qualitative numerical simulations for new displacement variables *x*_1_ and *x*_2_ and voltage *u*:

(2) $$\left\{\begin{array}{c} {\ddot{x}}_{1}+\delta_{1}{\dot{x}}_{1}-\delta_{2}{\dot{x}}_{2}+\eta_{1}x_{1}-\eta_{2}x_{2}+x_{1}\left(1-\frac{1}{\sqrt{1+x_{1}^{2}}}\right)+\theta u=\omega^{2}p\sin\left(\omega \tau \right),\\ {\ddot{x}}_{2}+\frac{1}{\mu }\delta_{2}{\dot{x}}_{2}+\frac{\eta_{2}}{\mu }x_{2}=\omega^{2}p\sin\left(\omega \tau \right)-{\ddot{x}}_{1},\\ \dot{u}+\sigma u-\vartheta {\dot{x}}_{1}=0. \end{array}\right.$$

where:

$$\begin{array}{c} \mu =\frac{m_{2}}{m_{1}},\;\eta_{1}=\frac{k_{1}}{k_{Z}},\;\eta_{2}=\frac{k_{2}}{k_{Z}},\;\delta_{1}=\frac{c_{1}+c_{3}}{\omega_{0}m_{1}},\;\delta_{2}=\frac{c_{2}}{\omega_{0}m_{1}},\;\omega_{0}^{2}=\frac{k_{Z}}{m_{1}},\\ x_{1}=\frac{q_{1}}{a_{0}},\;x_{2}=\frac{q_{2}}{a_{0}},\;\omega =\frac{\omega_{W}}{\omega_{0}},\;\tau =\omega_{0}t,\;p=\frac{A}{a_{0}},\\ \theta =\frac{k_{P}}{a_{0}m_{1}\omega_{0}^{2}},\;\vartheta =\frac{k_{P}a_{0}}{C_{P}},\;\sigma =\frac{1}{\omega_{0}C_{P}R_{Z}}. \end{array}$$

Based on such formulated mathematical model of the system, the results of model tests are presented in the following part of the paper. The simulations were carried out by using the MATHEMATICA differential equations solver with a variable time step and high precision.

## 3. Research Results

Table 1 summarizes the numerical values of the physical and geometric parameters of the tested design solution of the energy harvesting system.

The first stage of the research focused on assessing the impact of external load. The results of numerical simulations were visualized in the form of bifurcation diagrams, which were plotted against different values of the dimensionless excitation amplitude *p*. Bifurcation diagrams can be generated in several ways. The most popular among them is the identification of local minima and maxima of the time sequence. An analogous geometric structure of the bifurcation diagram is obtained by identifying the intersection points of the phase flow with the abscissa axis of the phase plane. Both approaches are simple, but it is not always possible to precisely determine the periodicity of the solution on their basis. For this reason, during the performed numerical experiments, we used an alternative approach based on Poincaré cross-sections. As a result, we can precisely define the periodicity of the solution in relation to control parameters. 

**Figure 2:**
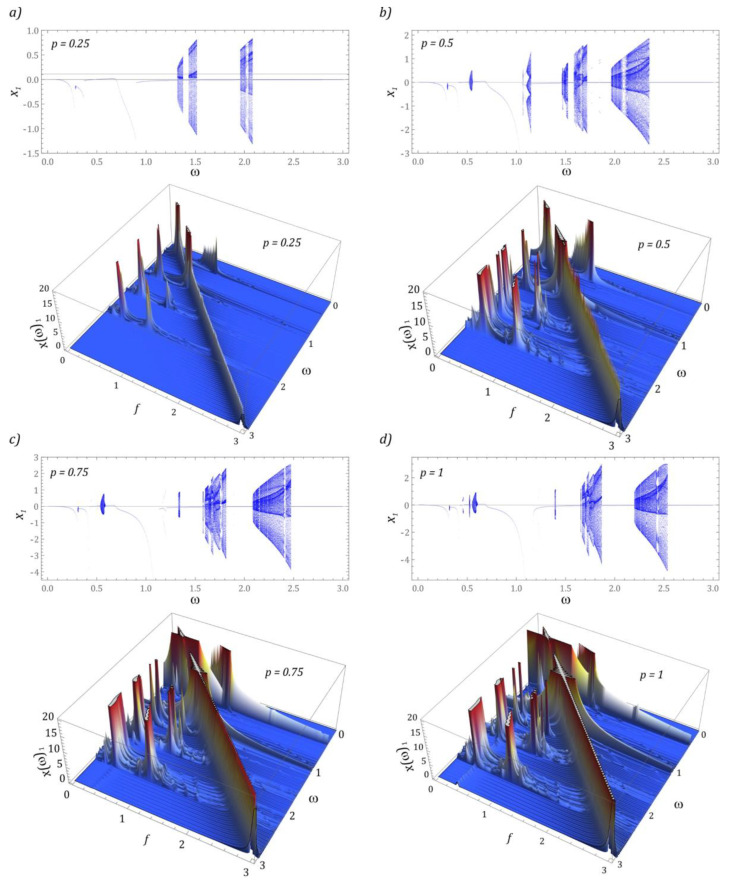
Bifurcation diagrams plotted against different levels of external dynamic load acting on the energy harvesting system: (**a**) *p* = 0.25, (**b**) *p* = 0.5, (**c**) *p* = 0.75, (**d**) *p* = 1. In the lower panels the corresponding frequency spectra are plotted (in terms of *f*) in 3*D* plots for each of *ω*.

In Figure 2 bifurcation diagrams are plotted against different levels of external dynamic load acting on the energy harvesting system. Additionally, for each *ω* the spectra of the responses are presented as functions of a frequency *f*. Based on the obtained results of the numerical simulations, it was found that with the increase in the dimensionless amplitude *p*, a shift of the areas of solutions with high periodicity towards higher values of the excitation frequency *ω* is observed.

Moreover, periodic solutions with a periodicity of 1 *T* dominate in the images of bifurcation diagrams. The dynamics of the energy harvesting system with a two-stage cantilever beam in a wide range of variability shows a complex nature, because at a low level of mechanical vibrations, relatively narrow zones of chaotic solutions appear in the bifurcation diagram (Figure 2a). Increasing the amplitude of mechanical vibrations *p* shifts the zones of chaotic solutions toward higher values of *ω*. Moreover, new zones of such solutions are excited in terms of low values of the dimensionless excitation frequency. The bands with periodicity doubling are very narrow. The only clear area in which this behavior of the system is observed locating in the *ω* ∈ [1.05, 1.15] band (Figure 2b). At the same time, it is worth paying attention to the fact that with an increase in the level of *p*, a narrowing or even complete extinction of the periodicity doubling zones is observed. As a result, the bifurcation diagrams show a rapid transition from the periodic solution to the chaotic one (Figure 2c). The situation is analogous to that shown in the diagram (Figure 2d).

One of the standard numerical tools used in the study of nonlinear dynamical systems is the Fast Fourier Transform [49,54]. It determines the nature of the solution we are dealing with. In the case of chaotic solutions, the harmonic component dominates in the amplitude-frequency spectra, which most often corresponds to the excitation frequency. Moreover, in the case of unpredictable solutions, a wide spectrum of harmonic components is excited, which are characterized by much lower amplitudes. Based on the drawn three-dimensional graphic images of the amplitude-frequency spectra, no excitation of a wide spectrum of harmonic components located in the vicinity of the excitation frequency *ω* was found.

The component representing the frequency of excitation prevails only in the case of periodic solutions. In the case of solutions with a large or very large periodicity, the low frequency dominates in the identified amplitude-frequency spectra. This character of the harmonic distribution may indicate the existence of quasiperiodic solutions. A precise answer to what nature of the solution we are dealing with is possible when additional detailed studies of the phase flows, and in particular, the analysis of Poincaré cross-sections are carried out. These issues are the subject of detailed analyzes included in Section 3.2.

### 3.1. Periodic Solutions

**Figure 3:**
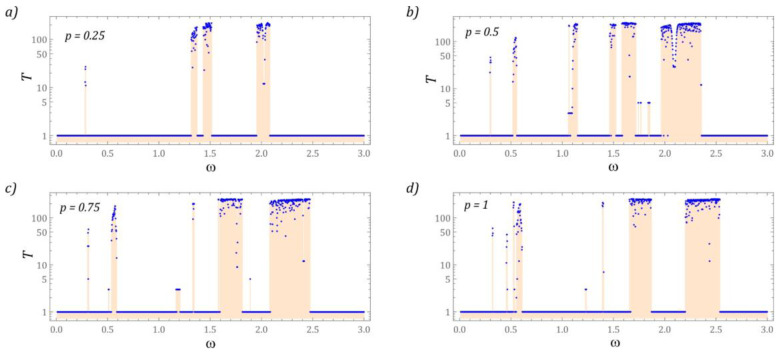
Periodicity of solutions of the tested energy harvesting system (note the log scale).

At this point, the focus was on the identification of periodic solutions that occur in the tested structure of the energy harvesting system. Periodic solutions gain significant importance because when the response of the system changes from periodic to chaotic, a reduction in the efficiency of energy harvesting is observed [53,56]. In our model tests, a numerical procedure based on the estimation of the distinct Poincaré points was used. The essence is to count the points of intersection of the phase flow with the control plane (Figure 3).

In the case of the acting on the system of mechanical vibrations of low intensity (Figure 3a), we are dealing with periodic solutions with a periodicity of 1 *T*. It is also possible to distinguish periodic solutions characterized by higher periodicity. However, the range of variability of *ω*, their occurrence is very narrow. A slightly wider band, corresponding to solutions approximately equal to 10 *T*, occurs in the vicinity of the frequency *ω* = 2. If the amplitude of the mechanical vibrations that affect the system increases to *p* = 0.5, then additional solutions with a periodicity of 3 *T* appear in the band *ω* ∈ [1.05, 1.2] and 5 *T* in the interval *ω* ∈ [1.7, 1.85] (Figure 3b). In this case, there are also 10 *T* periodic solutions, which are located at the end of the second zone of chaotic responses.

**Figure 4:**
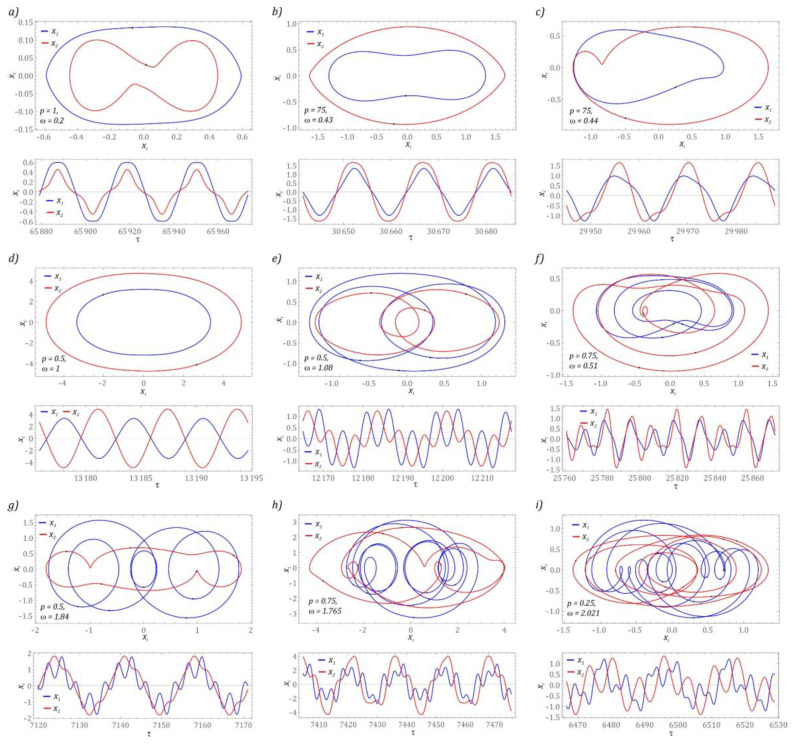
Graphical images of phase flows of periodic solutions. Phase portraits in the upper panes and time histories in lower panels. Cases (**a**–**i**) corresponds to the selected values of *p* and *ω* indicated in the figures.

In the periodogram presented on the graph (Figure 3c) there is a separate area of periodic solutions with a periodicity of 3 *T* in the *ω* ∈ [1.15, 1.2] band. These types of responses also take place in the range of low values *ω* = 0.5. On the other hand, in relation to 5 *T*-periodic solutions, a clear narrowing of the zones of their occurrence is observed. The 10 *T* periodic solution with respect to the case (Figure 3b) is absorbed by the very high periodicity response region. When the energy harvesting system is affected by high values of mechanical vibration amplitude *p* (Figure 3d), low-periodic responses are sporadic and point-like. It should be noted that a large “richness” of periodic solutions occurs in the range of low values. Examples of periodic solutions, illustrated in the form of phase trajectories and time series, are presented in the graphs (Figure 4).

In the range of low values of the dimensionless excitation frequency, there are periodic solutions with a periodicity of 1 *T*. The displacement of the second step of the flexible cantilever beam *x_2_* is smaller in relation to the displacement of the first step *x*_1_ (Figure 4a). This type of response is observed regardless of the value of the dimensionless amplitude of mechanical vibrations *p*, affecting the energy harvesting system. The influence of the *p*-parameter is visible in the form of a “refraction” of the phase trajectory at the moment of a velocity sign change. On the other hand, in the time domain, the shape of the *x*_1_ signal resembles a “flattened” sine wave. This “flattening” increases with increasing excitation amplitude *p* (Figure 4a). The same behavior of the system also takes place regarding the *x*_2_ coordinate (Figure 4b). It is worth mentioning here that with high displacement values of the second stage of the cantilever beam, it is possible to significantly improve the efficiency of energy harvesting. This claim is also justified by the synchronization of displacements of both steps of the cantilever beam. In the analyzed design, we also deal with asymmetric images of phase flows (Figure 4c). This nature of the system’s responses suggests the existence of a second solution with a similar efficiency of energy harvesting. Asynchronous vibration of both stages of the flexible cantilever beam occurs when the excitation frequency is close to unity (Figure 4d). In this case, it is also advisable to consider the possibility of placing an additional piezoelectric transducer on the second stage of the beam.

Periodic solutions with 3 *T* periodicity are in a relatively narrow range of variability of the dimensionless excitation frequency *ω* ∈ [1, 1.5]. Depending on the level of vibrations affecting the energy harvesting system, it is possible to distinguish two types of response. Similarly, to the 1 *T*-periodic solutions, this division is related to the vibration level of individual beam steps. From the point of view of topology, there are also two geometrical structures of the phase flows: symmetrical (Figure 4e) and asymmetrical (Figure 4f). Asymmetric 3 *T*-periodic solutions were identified in the range of low values of the dimensionless excitation frequency. It is worth mentioning here that the time responses of the displacement signals at higher levels of mechanical vibrations acting on the energy harvesting system are essentially synchronized.

In the tested model, periodic solutions with a higher periodicity *T* > 5 are very rare (Figure 4g). They are in very narrow bands between the zones of solutions with very high periodicity *ω* ∈ [1.5, 2] (Figure 2b). In the time domain, signals corresponding to the *5 T* periodic solutions are synchronized in phase. Graphical images of phase trajectories of periodic solutions with a periodicity of *9 T* are presented in the graphs (Figure 4h). The coordinate time series of the mathematical model also show the synchronous nature of the work. The system has a 12 *T* periodic response (Figure 4i), which is the only solution to have an even periodicity.

### 3.2. Chaotic and Quasiperiodic Solutions

**Figure 5:**
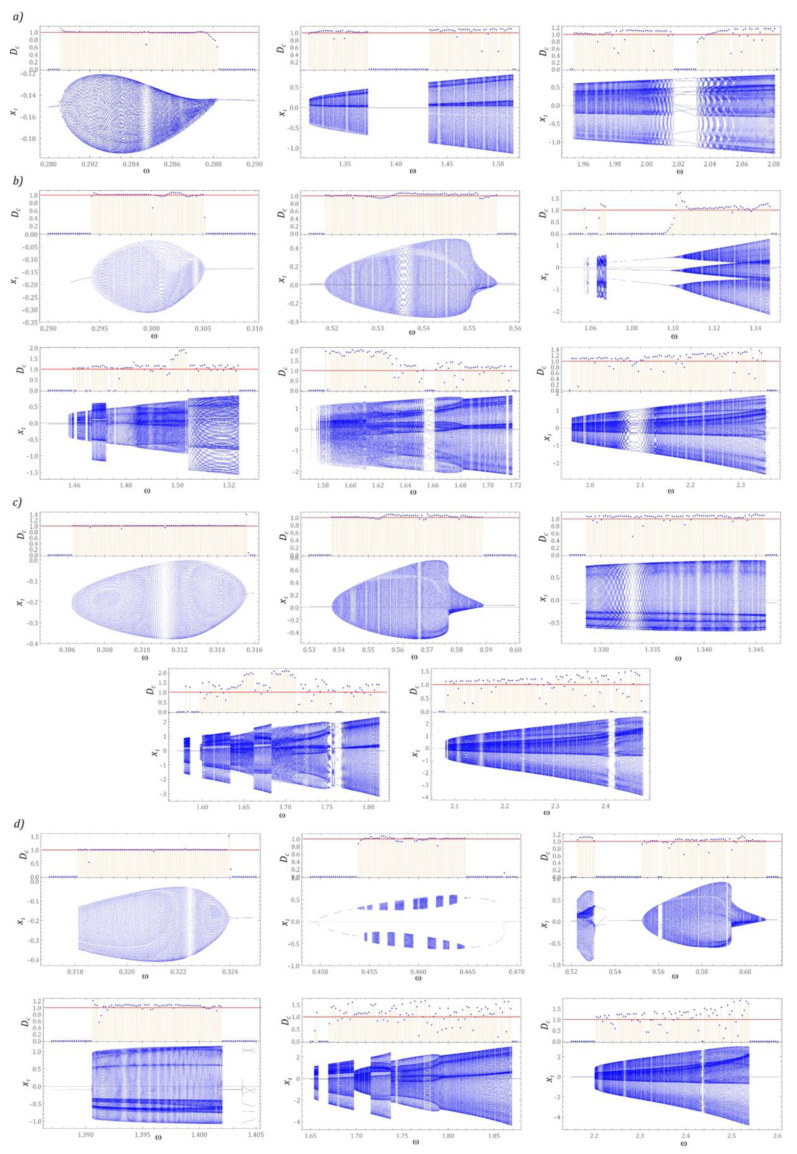
Solution zones with very high periodicity. Bifurcation diagrams and correlation dimension diagrams (see *D_C_* in the upper panels in the corresponding figures): (**a**) *p* = 0.25, (**b**) *p* = 0.5, (**c**) *p* = 0.75, (**d**) *p* = 1.

The subject of considerations in this subchapter are issues related to chaotic and quasiperiodic solutions. Based on the drawn bifurcation diagram, distinguishing a chaotic solution from a quasiperiodic solution is a difficult task. To give an unambiguous answer, additional numerical simulations are necessary, which boil down to plotting Poincaré sections and estimating the correlation dimension *D_C_*, by means of which the complexity of the plotted geometric structure of the Poincarè map is estimated. In the case of periodic solutions, the correlation dimension takes values close to zero. If we are dealing with quasiperiodic responses, then the values of *D_C_ ≈* 1. This value in the diagrams is marked with a straight line in red. With a view to reaching a compromise between the accuracy and time of numerical calculations, the values of the correlation dimension diagrams were identified in 100 cross-sections of solution zones characterized by very high periodicity. Moreover, the values of the *D_C_* coefficients were identified in relation to 5000 intersection points of the phase flow with the control plane. Such a large number of intersections was assumed due to the accuracy of the estimation of the correlation dimension. The next part presents the results of numerical simulations, which were visualized in the form of bifurcation diagrams drawn in magnification and many times greater resolution, and a diagram of the correlation dimension (Figure 5). As a result of this approach, we have a direct insight into the dynamics of the energy harvesting system, and above all, we have the ability to distinguish quasiperiodic solutions from chaotic ones.

**Figure 6:**
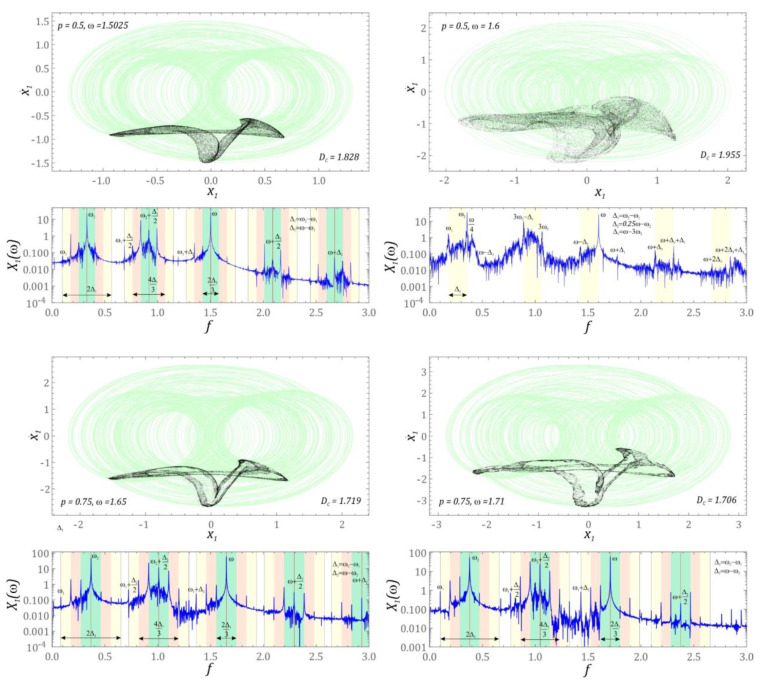
Geometric structure of chaotic attractors (**upper** panels) with amplitude–frequency spectra and dependencies necessary for identification (**lower** panels).

The distribution of the correlation dimension indicates that in the analyzed zones characterized by very high periodicity, quasiperiodic solutions dominate (Figure 5a). These solutions are separated by very narrow areas in which we deal with periodic motion. We deal with such dynamics of the energy harvesting system in the range of low values of the dimensionless excitation frequency *ω*. On geometric structures plotted on cross-sections, this manifests itself in the form of smooth curves. The presence of the occurring bends and distortions causes the correlation dimension to reach values of *D_C_* ∈ [1, 1.5]. In the plotted diagrams of the correlation dimension, *D_C_* < 1 values are noticeable. In these cases, the Poincarè sections are mapped with a point (Figure 6). Homogeneous zones of chaotic solutions occur when the energy harvesting system is affected by mechanical vibrations with amplitude *p* = 0.5 (Figure 5b) and *p* = 0.75 (Figure 5c). In these cases, the correlation dimension reaches values equal to approximately 2. If the mechanical vibrations affecting the tested energy harvesting system reach a high level, then in the frequency range ω < 1.5 we deal with periodic and quasiperiodic solutions (Figure 5d). Chaotic solutions occurring at such a level of mechanical vibrations affecting the system occur in the range of high values of the dimensionless excitation frequency ω > 1.5. Examples of graphic images of Poincaré cross-sections, representing the dynamics of the system, are presented in the graphs (Figure 6).

The presented examples of chaotic solutions are characterized by very high values of the correlation dimension *D_C_* ≈ 2, indicating a strongly non-linear behavior of the dynamical system. We are dealing with such solutions only when the energy harvesting system is influenced by external mechanical vibrations of the dimensionless amplitude *p* = 0.5 (Figure 5b) and *p* = 0.75 (Figure 5c). The identified Poincaré cross-sections of chaotic solutions show topological similarity, and the element that distinguishes them is the blur of the points of intersection of the phase flow with the control plane, which is visible in relation to the maps drawn for higher excitation frequencies. A direct comparison of the drawn maps clearly shows that the value of the *D_C_* increases with the blurring of the cross-section. The increase in the value of the correlation dimension is also directly related to the Fourier spectrum, which is characterized by the excitation of a wide spectrum of harmonics. For each case, the Fourier frequency amplitude spectrum was plotted.

Regardless of the characteristics of the input source that affect the energy harvesting system, the harmonic components dominate the Fourier spectra, which are a combination of the input frequency and two additional components *ω*_1_ and *ω*_2_. On their basis, causal relationships between them were identified. The analytical relationships constitute a formal basis for estimating the dominant frequency harmonics in the spectra. Note that in the case of “sharp” geometrical structures of Poincaré cross-sections, the distribution of the dominant bands in the amplitude–frequency spectrum is described by an analogous scheme representing the cause-and-effect relations between them. On the other hand, “blurred” Poincaré cross-sections, it is possible to identify only clearly dominant harmonics, since the entire spectrum is excited.

**Figure 7:**
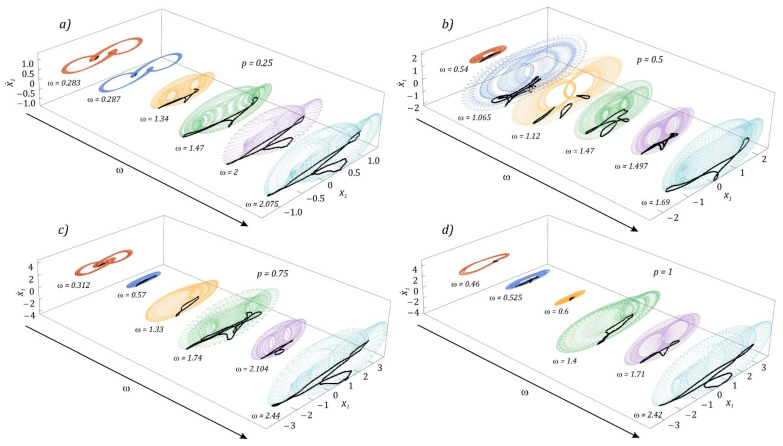
Evolution of quasiperiodic solutions (corresponding phase portraits with Poincarè sections): (**a**) *p* = 0.25, (**b**) *p*= 0.5, (**c**) *p* = 0.75, (**d**) *p* = 1.

The evolution of quasiperiodic solutions is presented below. The results of numerical calculations were visualized in the form of phase flows and Poincaré cross-sections plotted on them. To improve the legibility of the graphs, phase trajectories were drawn with colored dotted lines (Figure 7).

If the energy harvesting system is influenced by external mechanical vibrations with a dimensionless amplitude *p* = 0.25, then in the bands characterized by high periodicity of solutions, quasiperiodic responses dominate. There are two types of quasiperiodic attractors (Figure 7a). The first type occurs in the range of low values of the excitation frequency *ω* < 0.5, which resembles a deformed oval. On the other hand, the second one, an inclined letter T composed of closed curves, dominates in the field of high excitation frequencies. The highest number of quasiperiodic attractors is observed when the system is influenced by an external dynamic excitation with a dimensionless amplitude *p* = 0.5. The diagrams (Figure 7b) are limited only to illustrating the diversity of geometric forms, while the geometric structures of attractors occurring in the range of high excitation frequency values have been omitted. This was necessary because the graphic images of Poincaré cross-sections that appear there show topological similarity to the values of the excitation amplitude *p* presented in the graphs. In the example presented in the graphs (Figure 7c), we deal with four types of quasiperiodic attractors. The structures mapped with a deformed oval appear in the range of low values of *ω*. As it increases (*ω* = 1.33), bifurcation occurs, and the geometric structure of the attractor is mapped with two deformed ovals. A further increase in frequency forces bifurcation sequences, as a result of which at *ω* = 1.74 one of the more complex Poincare cross-sections is observed. It is worth mentioning here that in the vicinity of such quasiperiodic attractors, we deal with chaotic solutions. These attractors can, de facto, be treated as the basis of chaotic attractors.

**Figure 8:**
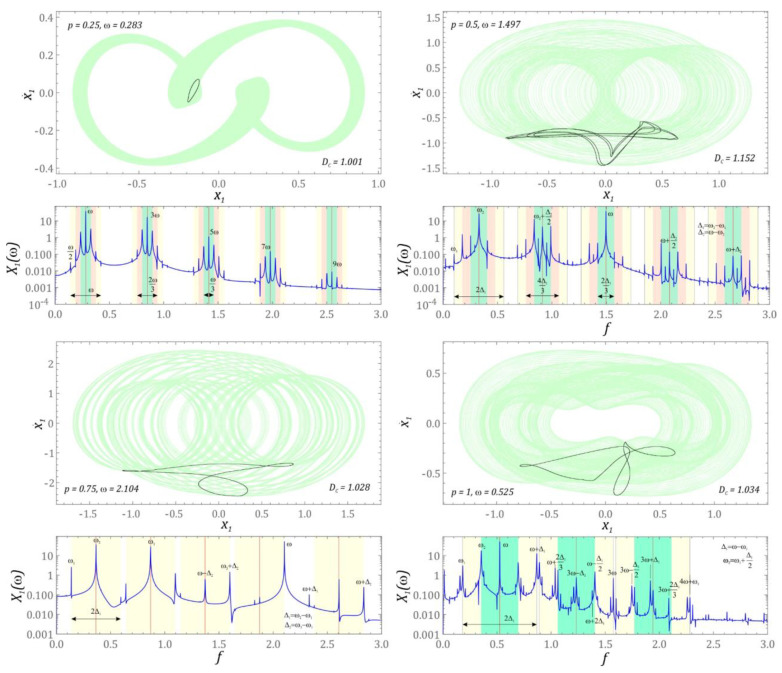
Geometric structure of quasiperiodic attractors with amplitude–frequency spectra and dependencies necessary for identification.

In the graphic images of the attractors, which were plotted for the amplitude *p* = 1, in principle, qualitatively new structures of the attractors do not appear. This situation occurs because we are dealing with basically the same bands of occurrence of solutions with high or very high periodicity but shifted in relation to the axis *ω*. As was the case with chaotic solutions, this time selected examples of quasiperiodic attractors were presented, with reference to which the harmonic distribution in the amplitude–frequency spectrum was analyzed (Figure 8).

Having in mind the most accessible analysis of the distribution of dominant harmonics in the amplitude-frequency spectrum, colored rectangles were used. For each rectangle, its width was defined, which was made dependent on the selected frequencies occurring in the Fourier spectrum. As a result, we could limit ourselves to providing only the characteristic frequencies occurring in the spectrum. In the case when the system is affected by the excitation with the characteristics *p* = 0.25 and *ω* = 0.283, the dominant harmonic components are in the vicinity of frequencies whose values are equal to the multiples of the excitation frequency *ω*. In the remaining examples, the values of the dominant harmonics are a combination of two or three excited harmonics in the spectrum. As was the case with chaotic solutions, the graphs also present the dependencies necessary for identification of these parameters.

### 3.3. Efficiency of Energy Harvesting

**Figure 9:**
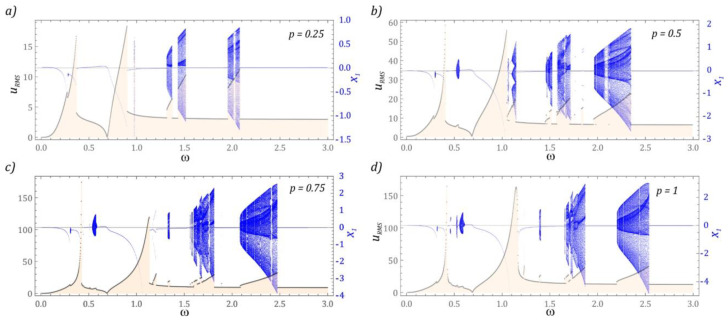
Diagrams of effective voltage values *u_RMS_* related to bifurcation diagrams *x_i_*: (**a**) *p* = 0.25, (**b**) *p* = 0.5, (**c**) *p* = 0.75, (**d**) *p* = 1.

Efficient energy harvesting is significantly dependent on the value of the voltage induced on the piezoelectric electrodes. In our investigation, the effective voltage value was adopted as the effectiveness indicator. For this purpose, a numerical procedure was developed to draw a diagram of the effective value of the voltage *u_RMS_* recorded on the piezoelectric in a wide range of variability of the dimensionless frequency of mechanical vibrations *ω* affecting the energy harvesting system. The plotted diagrams provide information on the efficiency of energy harvesting in relation to the set *ω* values. Considering the assessment of the impact of the solution on the efficiency of energy harvesting, the diagrams of RMS voltage values were referred to bifurcation diagrams (Figure 9).

The results of the obtained numerical simulations indicate that the highest effective voltage values occur in the range of low frequencies of mechanical vibrations affecting the energy harvesting system. It is worth noting that in the bands of occurrence of quasiperiodic solutions, a better efficiency of energy recovery is observed in relation to the neighboring periodic solutions. Greater efficiency of energy generation determined by the size of phase flows reflecting solutions in the zones of quasiperiodic responses. The limitation of the efficiency of energy harvesting in a system with quasi-zero stiffness is determined by the presence of a zone of chaotic solutions [53].

**Figure 10:**
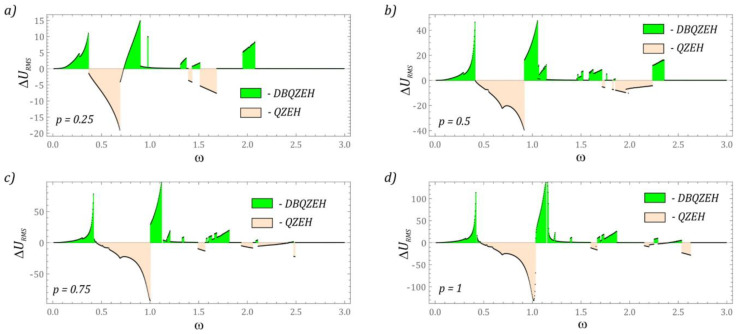
The difference of the effective values of the voltage induced on the piezoelectric electrodes mounted on the first stage of a flexible cantilever beam: (**a**) *p* = 0.25, (**b**) *p* = 0.5, (**c**) *p* = 0.75, (**d**) *p* = 1. DBQZEH–double-beam quasi-zero-stiffness energy harvester, original QZEH–quasi-zero-stiffness energy harvester (see [53]).

The next part presents the results of numerical simulations showing the efficiency of energy generation of the considered structural solution in relation to the system without an additional step of the cantilever beam. The difference in the RMS voltage Δ*U_RMS_* recorded on the electrodes of their piezoelectric elements was adopted as a measure of the evaluation of the energy recovery capacity of two different design solutions. The results of the model tests were illustrated in the form of diagrams (Figure 10), where positive values indicate a better energy harvesting efficiency of the analyzed design solution (DBQZEH). On the other hand, negative values show that the single-stage cantilever beam (QZEH) system shows better energy harvesting efficiency. If Δ*U_RMS_* = 0, then both design solutions are characterized by similar efficiency of energy generation.

The results clearly show that in the range of low frequency values *ω* < 0.5 of mechanical vibrations that affect the energy harvesting system, the solution with a two-stage cantilever beam is more efficient. It should be noted that this statement applies to all external excitation amplitudes *p*. In the band located between the resonances of the system with a two-stage cantilever beam, the design solution with a single stage shows a much better efficiency of energy generation. At this point, we do not precisely define the width of the band located between the resonances, because its position moves toward higher values of ω with increasing amplitude of the external excitation *p*.

**Figure 11:**
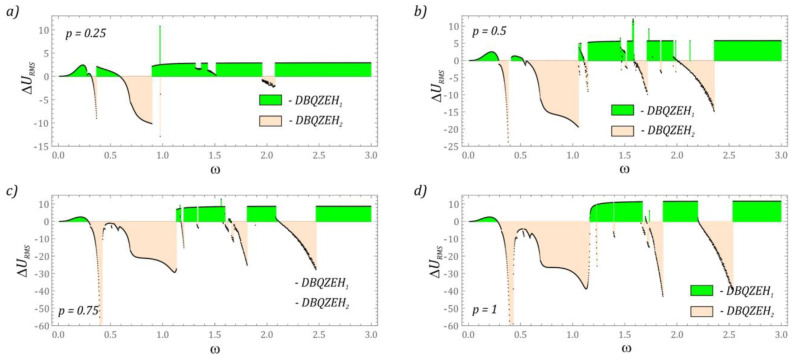
The difference of the effective values of the voltage induced on the piezoelectric electrodes mounted on the first or second stage of a flexible cantilever beam: (**a**) *p* = 0.25, (**b**) *p* = 0.5, (**c**) *p* = 0.75, (**d**) *p* = 1. DBQZEH_1_–double-beam quasi-zero-stiffness energy harvester (piezoelectric on the first beam), DBQZEH_2_–double-beam quasi-zero-stiffness energy harvester (piezoelectric on the second beam).

A legitimate question is how the location of the piezoelectric element affects the efficiency of energy harvesting. For this reason, the results of numerical experiments comparing the efficiency of energy generation when the piezoelectric was mounted on the first and second stages of a flexible cantilever beam are presented below. The results of the numerical experiments were visualized in the form of diagrams of the difference of the RMS values Δ*U_RMS_*. In the plotted diagrams, the subscript value represents the attachment point of the element that transforms mechanical energy into electrical energy. The value 1 is assigned to the first stage and 2 to the second. The obtained results of computer simulations showing the magnitude of the voltage induced at individual stages were visualized in graphs (Figure 11).

The results of numerical simulations (Figure 11a) show that in the range of low *p* amplitudes, it is more efficient to obtain energy by installing a piezoelectric on the first stage of the cantilever beam. Installing a piezoelectric on the second stage is characterized by a better energy efficiency in relatively narrow bands *ω* ∈ [0.3, 0.4], *ω* ∈ [0.6, 0.9] and in the vicinity of *ω* = 2. With the increasing value of the dimensionless amplitude *p*, the amount of energy generated on the second stage of the beam is increased, and regardless of the value of *p*, it is more advantageous to install a piezoelectric on the second stage in the range of very high excitation frequencies *ω* > 2.5. Increasing the amplitude *p* to the level of 0.5 (Figure 11b) broadens the effective energy generation in the *ω* ∈ [0.5, 1.1] zone, with the amount of energy harvested essentially remaining constant.

Only in the vicinity of the second resonance is an improvement in the efficiency of energy recovery in relation to the middle part of the band. When the amplitude *p* reaches the value of 0.75 (Figure 11c), the first two bands defining the efficient energy harvesting of the piezoelectric elements mounted on the second stage are combined. With the further increase in mechanical vibrations affecting the energy harvesting system, the situation basically does not change. The only important elements distinguishing the diagrams (Figure 11c,d) are the values of the effective voltage registered on the piezoelectric electrodes.

**Figure 12:**
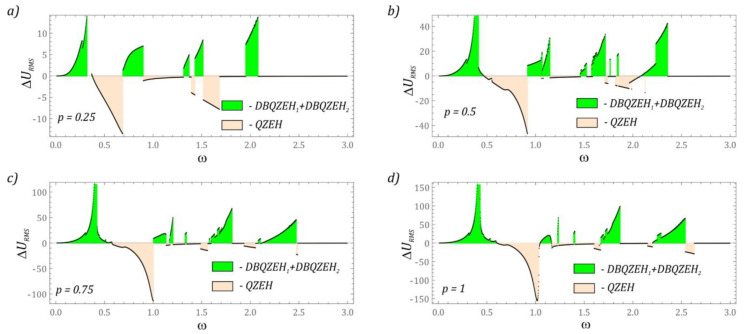
The difference of the effective values of the voltage induced on the piezoelectric electrodes mounted on the first and second stage of a flexible cantilever beam: (**a**) *p* = 0.25, (**b**) *p* = 0.5, (**c**) *p* = 0.75, (**d**) *p* = 1. DBQZEH_1_–double-beam quasi-zero-stiffness energy harvester (piezoelectric on the first beam), DBQZEH_2_–double-beam quasi-zero-stiffness energy harvester (piezoelectric on the second beam), QZEH–quasi-zero-stiffness energy harvester.

The last element of the research was the assessment of the efficiency of energy generation in a system with piezoelectric elements mounted on both steps of the cantilever beam. The identified RMS voltage values were related to the system with a single-stage cantilever beam (Figure 12). It is worth noting that regardless of the level of vibrations affecting the energy harvesting system, there are still *ω* zones in which the system with a single-stage cantilever beam demonstrates better energy harvesting efficiency. However, in terms of higher values of *ω*, we are dealing with an increase in the efficiency of energy generation. In relation to the results presented in the diagram (Figure 9), the efficiency in the vicinity of the second resonance is limited. Such a situation should be seen with asynchronous vibrations of both steps of the cantilever beam. Asynchronous movement of individual stages can be minimized by applying voltage signals to rectifier bridges, and only then summing.

## 4. Final Conclusions

The results of numerical simulations show that the considered levels of mechanical vibrations, periodic and quasiperiodic solutions dominate, while chaotic responses are rare. In the zones of quasiperiodic responses, an increase in the effective value of the voltage recorded on the piezoelectric electrodes is observed. In the tested system, a sharp reduction in the efficiency of energy harvesting in some bands is determined by the second stage of a flexible cantilever beam, which acts in the first stage as a dynamic vibration eliminator.

Note that, taking the composite two-stage beam, we extended the results with respect to the previous studies on simple single-stage quasi-zero-stiffness energy harvesters. In the limit of the composite beam uniformity, the new results coincide with previous study for the harvester with a single-state beam resonator.

Considering effective energy harvesting, it is advisable to attach piezoelectric energy transducers to each step of the flexible cantilever beam. However, this conclusion is the product of a case study. Therefore, in the next step of our research, we plan to manipulate the system parameters to provide conditions for the displacement amplifier proposed in [54]. Moreover, coexisting solutions will be studied more carefully by sampling the initial conditions to estimate their stability. We are also planning experimental verification of the numerical results.

## Figures and Tables

**Table 1:** Geometric and physical parameters of the model.

Name	Symbol	Beam I	Beam II
Length	*L_i_*	0.13 m	0.06 m
Width	$b_{i}$	0.025 m	0.018 m
Height	*h_i_*	0.0003 m	0.0002 m
Material	*E*	210 GPa	
Load mass	*m_i_*	0.025 kg	0.026 kg
Stiffness	*k_i_*	16 Nm^−1^	35 Nm^−1^
Energy dissipation	*c_i_*	0.00013 Nsm^−1^	0.00019 Nsm^−1^
Length of the compensation springs	*a_0_*	0.03 m	
Stiffness of the compensation springs	*k_Ci_*	72 Nm^−1^	
Total resistance	*R_Z_*	1.1 × 10^6^ Ω	
Piezoelectric capacity	*C_P_*	72 nF	
Electromechanical constant of piezoelectric converter	*k_P_*	3.985 × 10^−5^ m/V	

## Data Availability

Data are contained within the article.
